# Impact of Orthodontic Treatment on Periodontal Tissues: A Narrative Review of Multidisciplinary Literature

**DOI:** 10.1155/2016/4723589

**Published:** 2016-01-19

**Authors:** Angelina Gorbunkova, Giorgio Pagni, Anna Brizhak, Giampietro Farronato, Giulio Rasperini

**Affiliations:** ^1^Department of Biomedical, Surgical and Dental Sciences, University of Milan, Milan, Italy; ^2^UOC Maxillofacial and Dental Surgery, Foundation IRCCS Ca' Granda Polyclinic, 20142 Milan, Italy

## Abstract

The aim of this review is to describe the most commonly observed changes in periodontium caused by orthodontic treatment in order to facilitate specialists' collaboration and communication. An electronic database search was carried out using PubMed abstract and citation database and bibliographic material was then used in order to find other appropriate sources. Soft and hard periodontal tissues changes during orthodontic treatment and maintenance of the patients are discussed in order to provide an exhaustive picture of the possible interactions between these two interwoven disciplines.

## 1. Introduction

Thanks to the increasing demand in appearance, orthodontic treatment is being more and more adopted in the adult population. As adult orthodontic patients may also have restorative and periodontal needs, the interaction between different specialties becomes even more important. Many periodontal patients may present with pathological tooth migration or other deformities where orthodontics may represent an important part of their treatment. Both periodontists and orthodontists should understand the results of one's work on the other's and cooperate in clinical practice to deliver the best possible treatment to their patients.

**Figure 1:**
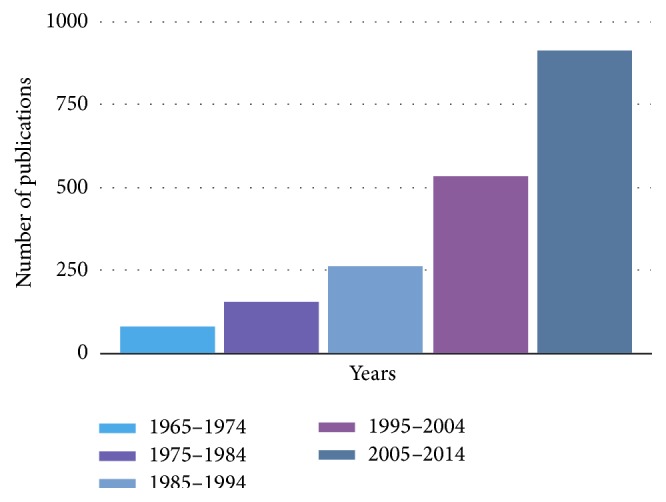
Increasing number of data observing orthodontics with periodontology reflects the increasing interest in multidisciplinary approach with time.

The number of publications evaluating orthodontics and periodontal interactions keeps increasing (Figure 1). The number of papers published in the last 5 years equals that of those published in the previous 10 years (2000–2010), which, in turn, almost equals the one of the previous 60 years (1940–2000).

The aim of this review is to explore this vast body of literature, select specific critical concepts and multidisciplinary connections, and highlight the importance of specialties cooperation.

An electronic database search was carried out using PubMed abstract and citation database with the keywords: “periodontology” AND “orthodontics” published in English. Reviews, clinical trials, animal studies, comparative studies, evaluation studies, and case reports were selected. Two authors, Angelina Gorbunkova and Anna Brizhak, selected the papers. Bibliographic material from the papers was then used in order to find other appropriate sources.

Observations of soft and hard periodontal tissues' changes during the orthodontic tooth movement (OTM) in orthodontic and periodontal literature will be described.

## 2. Soft Tissue Changes

Orthodontic treatment can be implemented to improve dental aesthetics not only by correcting position of the jaws and deformities of dentition, but also by creating the conditions for improved gingival health. Adult patients previously affected by periodontal disease often present with “black triangles” due to missed interdental papillae height. By means of orthodontics, it is possible to correct teeth position and to improve soft tissue aesthetics. It was suggested that orthodontic teeth approximation might change the topography of the interproximal alveolar crest level and enhance the position of the interdental papilla [1] although black triangles may also appear as a consequence of teeth alignment when resolving crowding. Tarnow et al. in 1992 [2] and Wu et al. in 2003 [3] suggested that the filling of the interdental space with the papilla could be determined by the position of the contact point with respect to the bone crest position. Tooth reshaping may help moving the contact point more apically during orthodontic teeth approximation which might help to achieve good aesthetic results in the interdental area [4].

It should however be taken into consideration that during OTM some adverse effects on the soft periodontal tissue may be observed. The most frequently occurring changes in soft tissues are gingival overgrowth (GO), gingival recessions (GR), and gingival invaginations (GIs), which commonly occur in orthodontic extraction cases.

Gingival overgrowth is a very common condition in the orthodontic population that is characterized by gingival enlargement possibly resulting in pseudo-pocketing with or without attachment loss. When involving the anterior region, it may have an impact on oral health-related quality of life [5]. Traditionally, GO was considered as an inflammatory reaction consecutive to bacterial plaque accumulation [6]. Other factors as chemical irritation produced by materials used for banding, mechanical irritation by bands, and food impaction have been suggested to explain the pathogenesis of GO [7]. In 1972, S. Zachrisson and B. U. Zachrisson [8] had reported gingival enlargement in patients maintaining excellent oral hygiene. More recently, Şurlin et al. [9] evaluated orthodontic patients with good dental hygiene exhibiting GO without any clinical signs of gingival inflammation. These patients exhibited elevated matrix metalloproteinase-8 (MMP-8) and matrix metalloproteinase-9 (MMP-9) levels in gingival crevicular fluid (GCF). It was considered that, during orthodontic treatment, the mechanical stress appeared to be one of the key factors determining the increase of MMP-9 production and the onset of GO. Some authors also evaluated the possible role of an allergic reaction to nickel, releasing from the orthodontic appliances made of stainless steel. In vitro and in vivo studies suggest that released nickel ions may cause an exposure time dependent allergic reaction characterized by an upregulated proliferation of keratinocytes and increased epithelial cell proliferation [10, 11]. It may be therefore important to treat patients with nickel-free appliances and to adopt questionnaires to evaluate previous history of allergies to metals as they have been linked to an increased frequency of GO [12–14].

Enlargement of interdental papillae and accumulation of gingival tissue may appear due to the application of compressive or retraction forces at the site of extraction space closure. In orthodontic treatment, the extraction of teeth, most commonly, first or second premolars, may be required. Orthodontic space closure of extraction sites may result in gingival invagination or accumulation of gingival tissue [15].

Gingival ingrowth was defined by Robertson et al. [16] as a linear invagination of the interproximal tissue with mesial and distal orientation and an intragingival probing depth of at least 1 mm. The frequency of GI is reported to be high and may be observed more often in the lower jaw [16–18]. Due to its location, GI may render adequate plaque control complicated, possibly contributing to gingival and periodontal disease occurrence [16, 19]. There is a correlation between gingival cleft and timing of OTM. Significantly more GIs were reported when there was a delay in space closure and orthodontic treatment was initiated late after tooth extraction [17, 20]; therefore, proper communication between specialists is particularly important. Gingival ingrowth may exhibit a high degree of variability, ranging from a minor superficial crease in the gingiva to severe defects with complete penetration of the alveolar ridge (25% of all clefts) [17]. According to the GI severity, treatment strategies may vary. When GI is located in soft tissues only, it may be treated using a cold blade or the electric cautery with no significant difference between the two gingivectomy techniques [21]. Soft tissue diode laser in the management of mucogingival problems may present some advantages because of the minimal postoperative pain reposted with the use of these devices [22]. To prevent GI formation during OTM in the postextraction area, guided bone regeneration (GBR) can be applied; however, the best timing for tooth approximation to be initiated after surgery is still under discussion [23–25].

Both orthodontic and periodontal literature have thoroughly discussed gingival recession that may lead to unsatisfactory aesthetics, root sensitivity, increased susceptibility to caries, tooth abrasion, and following difficulties in maintenance of oral hygiene. OTM may either promote GR formation or improve soft tissue conditions [26–32]. Among orthodontic patients, up to 10–12% exhibited gingival recessions [26, 33]. One of the main reasons for GR development is believed to be a continuous mechanical trauma by toothbrush [34, 35], but Matthews [36] and Rajapakse et al. [37] suggested that there is no good evidence of direct link between toothbrushing and appearance of noninflammatory GR. Several anatomical and morphological characteristics were suggested to play a role in GR formation. During OTM, alveolar bone dehiscences may occur when tooth roots move through the alveolar cortical bone [38–40]. More often, this type of movement is carried out in patients with a small alveolar process, thin buccal or lingual bone plates, eccentric position of teeth, basally extended maxillary sinus, and progressive alveolar bone loss [41]. It should be noted that if the tooth is moved within the envelope of the alveolar bone, the risk of harmful side effect on the marginal soft tissue is minimal [40, 41]. The direction of applied orthodontic forces may also have an impact on soft tissues. Some studies suggested that controlled proclination of mandibular incisors could be carried out in orthodontic patients with no risk of periodontal breakdown if good level of dental hygiene is provided [27, 33, 42, 43]. Recent studies suggested that [44, 45] proinclination orthodontic movement may be significantly associated with a reduction of the keratinized tissue width. These findings are supported by other previous studies suggesting that labial tooth movement may result in decreased buccolingual tissue thickness and reduce the height of the free gingiva facilitating GR. On the other hand, lingual tooth movement may have the opposite effect [29, 38, 44]. Periodontal biotype also has been suggested to be an important factor in GR development. A strong correlation was found between thin biotype and proinclination orthodontic movement in terms of GR depth and keratinized tissue width. In contrast to patients who performed a thick gingival biotype, those with a thin-scalloped biotype are considered at risk [44, 45]. Thin periodontal biotype and amount of attached gingiva were found to be significantly related to labial plate thickness and alveolar crest position. Thin periodontium demonstrates decreased resistance to mechanical stress or inflammation and may correlate with development of GR [28, 42, 45, 46]. In light of this, an accurate evaluation of gingival thickness before starting OTM is definitely recommended [44, 45].

As for any condition with multifactorial etiology, it is important to weight the importance of any contributing factor to evaluate patient predisposition prior to initiating therapy. Because of this, we recommend critically evaluating each specific case before coming to a definitive treatment plan. Patient-related factors may also play an important role in the decision making process.

While awaiting more evidence-based information on how to proceed in different case scenarios, we would like to provide our personal opinion in order to highlight areas of interest for possible future research.

Mucogingival surgery during orthodontic treatment aims to change soft tissue characteristics in order to create more favourable conditions for the mechanical stress resistance. Nevertheless, improved gingival characteristics may not guarantee the absence of gingival recession after OT especially when significant dental arch expansion or labial proclination is performed and a second surgery may be needed after the end of orthodontics.

Our insight when evaluating orthodontic cases at risk for possible GR is that patients with a thin biotype should receive soft tissue grafting prior to OTM in order to reduce the risk and the extent of the possible GR. Thus far, it is not clear which gingival and movement characteristics may predispose to GR and what would be the incidence of GR in each specific scenario. The efficacy of preventive surgeries should also be further analysed: in example, we would like to know the number of preventive surgeries in correspondence with the number of patients that would actually develop GR. We would also like to know how many patients receiving a preventive surgery will also require a second corrective surgical procedure.

A different scenario can be found, should GR occur during OTM. In these cases, soft tissue grafting is indicated and should be performed as soon as possible given that all other parameters (gingival inflammation, trauma, etc.) are controlled. The aim is to treat the recession once it is still minimal and improve treatment prognosis. Orthodontic therapy should be carefully evaluated in this period of time in order to determine whether to stop or to slow down OTM until wound healing is complete. Clearly, the timing of appearing of the GR is important and we should better understand the implications of a GR occurring in the initial third of orthodontic treatment versus close to the end of OTM.

When preexisting GRs are found before orthodontic treatment, the impact of orthodontic treatment should be carefully evaluated. Should the tooth be planned for lingual tooth movement, mucogingival surgery may not even be required and OTM alone may end up treating or at least not aggravating the recession. When necessary, the prognosis of mucogingival surgery may be improved after the tooth is moved lingually. Should the tooth be moved labially instead, a corrective mucogingival procedure aiming to avoid disease progression should be taken into consideration. OTM may be initiated once wound healing is complete (3-4 months). At the end of orthodontic therapy, the site should be reevaluated and a second intervention may be needed in limited cases.

Every clinical case may include a combination of different predisposing and precipitating factors that can affect the treatment outcome; therefore, it is important to evaluate risk factors while planning orthodontic treatment in order to avoid undesired consequences of the delivered therapy. Risk management is possibly the most important factor when treatment planning these patients. We encourage researchers to further evaluate unclear aspects such as patient/tooth/site predisposition to gingival recession and ideal type and timing of treatment and to generate incremental systems for hierarchical clustering, which would be able to put together different probabilistic nodes in the determination of specific clinical solutions.

## 3. Bone Changes

Mechanical force during OTM results in bone resorption and bone apposition widely discussed in both orthodontic and periodontal literature. In health, during OTM, all components of periodontal attachment apparatus, including the osseous structure, periodontal ligament, and the soft tissue components, move together with the tooth. The same applies to patients with reduced but healthy periodontal tissues [32, 47, 48]. After periodontal treatment, light orthodontic forces combined with good dental hygiene control may be enough to result in teeth alignment when periodontal support is reduced.

OTM in presence of intrabony pockets presents a different challenge for clinicians. Several studies suggested that OTM after surgical periodontal treatment may have an impact on the morphology of bone defects, decrease pocket depth, and enhance connective tissue healing. All the positive changes in supporting apparatus were achieved only when a good dental hygiene control had been implemented. Some authors applied intrusive orthodontic forces and reported clinical and radiological improvements [49, 50]. Also, it was reported in histological study by Melsen et al. [51] that new cementum formation and new collagen attachment may be obtained by orthodontic intrusion in presence of good dental hygiene. da Silva et al. [52] in their study on dogs intruded teeth with furcation defects and suggested that class-III defects may be clinically eliminated or reduced resulting in clinical attachment level gain. Another study investigated the influence of tilting movements in presence of intrabony pockets and reinforced the conclusion that OTM may be performed in teeth with bone defects without damage to the periodontal attachment level [53]. Polson et al. [47] further evaluated the attachment apparatus on such teeth and reported the presence of long junctional epithelium between the bone and the root surface after teeth movement into and through the defect, suggesting no regeneration from the supporting apparatus. Therefore, it was recommended to apply GTR techniques in the treatment of intrabony defects before orthodontic therapy in order to achieve regeneration instead of repair.

Effectiveness of periodontal regeneration in the treatment of intrabony defects is well documented and supported by histological studies. All the benefits of guided tissue regeneration (GTR) may be maintained over a long period of time (over 10 years) [54, 55]. It is commonly believed that bony pocket topography is important for the prognosis of the regenerative treatment; however, a recent systematic review claimed clinical outcomes of periodontal regeneration to be influenced by patient behaviors and surgical approach more than by tooth and defect characteristics [56]. The combined adoption of orthodontic therapy and periodontal regeneration of teeth with infrabony defects may be suggested in multiple situations. Orthodontic extrusion, intrusion, and sagittal tooth movements with different timing of OTM after GTR were described in the literature. Evaluating apical downgrowth of junctional epithelium, Nemcovsky et al. [57] suggested that periodontal regeneration might be indicated prior to OTM. In 2003, Diedrich et al. [58] performed a study on orthodontic intrusion and translation of teeth with 3-wall bony defects previously treated with open flap debridement combined with enamel matrix protein. In the intrusion group, a slight epithelial downgrowth, extensive cementogenesis, and bone apposition were documented leading to results comparable to those noted on the tension site of translation group. Defects on the pressure side were additionally covered with resorbable membrane and after OTM showed markedly reduced bone apposition. These results may indicate the possible influence of biomaterial degradation on regenerative outcomes, which was also suggested in other studies [52, 59]. Araújo et al. [60] suggested that it was possible to move teeth into areas previously augmented with biomaterial. Orthodontic forces were applied 3 months after grafting and no impediment in OTM was observed. Some authors suggested that the optimal timing to begin OTM after GTR is 4 weeks after surgery when mitotic activity of periodontal cells is increased and OTM occurs in immature bone [52, 61]. Attia et al. [62, 63], evaluating the effectiveness of different timing for initiating active orthodontic treatment after GTR, suggested that significant improvements of periodontal regeneration may be observed in defects treated with the immediate application of orthodontic forces after surgery. Others demonstrated that orthodontic treatment provided 1 year after GTR, when both hard and soft tissues are mature, caused no detrimental effect on periodontal regeneration outcomes [4]. Taking into account the results of these different studies, it may be concluded that several factors such as direction of tooth movement and timing and choice of biomaterials should be taken into consideration during treatment planning, although more well-designed clinical trials are needed to further clarify the mechanisms involved with wound healing when orthodontic forces are applied [64].

## 4. Maintenance

Our initial search included a large amount of studies evaluating the effects of different levels of oral hygiene in patients undergoing OTM. This highlights the importance of maintenance in dental practice especially in cases where orthodontics is combined with periodontal treatment. For all patients undergoing orthodontic treatment on fixed appliances or wearing fixed retainers, it is difficult to maintain a good level of oral hygiene, because orthodontic constructions and accessories may hinder conventional brushing and flossing. Meanwhile, deficient oral hygiene in orthodontic patients appears to be a key factor in the development of white spot lesions, dental caries, and gingival inflammation due to the presence of dental plaque accumulation [65, 66]. In the presence of insufficient dental hygiene, orthodontic treatment may lead to the transposition of the supragingival dental plaque subgingivally resulting in infrabony pocket formation [67].

The type of appliance (fixed or removable), bracket material, bonding technique (lingual or buccal bonding), and type of retainer selected for orthodontic therapy may all influence the patient ability to maintain a good level of plaque control. During OTM oral malodor, plaque index and gingival index increase and first changes may be observed immediately after bonding [66, 68].

Some authors suggested dental plaque accumulation in patients wearing fixed appliances to be greater than that in patients wearing removable appliances [69]. While the evidence supporting this sentence is not that strong and more well-designed clinical trials should be carried out in order to investigate clinical parameters of periodontal status in two different treatment modalities, clinicians may want to consider this piece of information when treatment planning periodontal patients for orthodontic therapy. Lingual orthodontic appliances showed higher plaque retention compared to labial orthodontic appliances due to more difficult access for daily maintenance [70]. Despite the fact that there was no significant difference in plaque accumulation with regard to the type of ligation [71, 72], bracket material also seems to influence quantity and location of plaque accumulation. Stainless steel brackets appeared to harvest significantly bigger amount of plaque when compared to ceramic, sapphire, and polycarbonate brackets. When using ceramic brackets, the greatest amount of plaque was shown to accumulate on occlusal and gingival surfaces, while mesial and distal surfaces were shown to accumulate more plaque when adopting stainless steel brackets [73–75]. In addition, stainless steel surfaces were suggested to attract less biofilm than gold [76–78]. The presence of fixed retainers may be associated with a risk of higher level of plaque accumulation, gingival recession, and bleeding on probing. Patients with multistrand wire retainers exhibited more plaque accumulation on the distal surfaces of the lower anterior teeth in comparison with a single span, round wire retainers [79, 80].

In order to reduce risks of periodontal breakdown during and after OTM, more attention should be paid to the orthodontic devices' characteristics while planning. Periodontal status in orthodontically treated patients might be assessed not only during therapy and after debonding, but likely also during follow-ups in retention period. In periodontal patients undergoing orthodontic therapy, plaque control has to be closely monitored.

Numerous articles extensively discussed advantages and disadvantages of different types of toothbrushes: manual toothbrushes, sonic, orthodontic, powered, oscillating-rotating, ultrasonic, and ionic [81–84]. According to the recent update of a Cochrane review [85] based on 51 articles with a total of 4624 participants, it was suggested that powered toothbrushes may provide a significant benefit when compared with manual toothbrushes. Several studies in orthodontic patients also supported these findings and demonstrated higher effectiveness of oscillating-rotating toothbrushes in dental plaque removal and gingivitis reduction when compared to manual brushes [86, 87]. It should be taken into consideration that patients' motivation and repeated oral hygiene instructions may be a crucial factor for patients undergoing OTM with fixed appliances [88, 89]. Motivation of orthodontic patients may include different educational techniques: oral hygiene instructions, showing images of possible complications, the use of plaque-disclosing tablets, demonstrations of brushing techniques on models, and even showing patients phase contrast microscopy of their plaque samples [90, 91].

Orthodontic patients who are not able to establish satisfactory oral hygiene levels are recommended to receive some additional aids such as dental varnishes, gels, mouth washes, or dentifrices. Chlorhexidine (CHX) which may be included in different kinds of vehicles shows antibacterial effectiveness against gingival inflammation and cariogenic bacteria and may also reduce the severity of traumatic ulcers during OTM [92–96]. The discussion on the side effects related to long-term use of CHX such as tooth staining is commonly debated and is considered to be related to its concentration. By using mouthrinses and dentifrices with lower concentrations of CHX, it is possible to reduce tooth decoloration without significant difference in reduction plaque formation and gingival inflammation [96, 97]. The inclusion of fluoride in CHX dentifrices may help in providing better prophylaxis of white spot lesion formation while simultaneously reducing gingival inflammation [98].

Patient's compliance, motivation, and oral hygiene maintenance are universally recognized as important factors when evaluating the impact of OTM on their periodontal status. These parameters are important for maintaining the periodontal condition after nonsurgical and surgical periodontal therapy and should be continued afterwards. Taking into account additional difficulties in daily dental hygiene for orthodontic patients during treatment with fixed appliances, regular monitoring of adults with predisposition for periodontal breakdown during OTM is mandatory. Orthodontists should pay great attention to the dental health education, emphasizing oral hygiene instructions and regular periodontal care. Periodontal check-ups and good quality professional hygiene maintenance appointments are essential even after the completion of orthodontic treatment. In other words, periodontal maintenance should be provided from the beginning of periodontal therapy, through all the steps of periodontal treatment, it should be even more closely monitored during orthodontic treatment, and it should be continued throughout the lifetime of the patient.

## 5. Conclusions

Well-coordinated multidisciplinary dental treatment aims to provide satisfactory aesthetics, function, and long-term prognosis for patients. An effective cooperation makes it possible to observe clinical problems from different perspectives and to better understand the interactions between different specialties. Periodontal health is essential for any form of dental treatment. In order to avoid undesirable consequences during and after OTM, a thorough assessment of periodontal health should be provided. Attention should be paid to dental hygiene parameters especially in patients wearing fixed appliances and in periodontally susceptible individuals.

In this review, we elected some clinical aspects where periodontal and orthodontic knowledge come together to provide a more exhaustive picture of the orthodontic treatments impact on periodontium. We discussed possible effects of OTM on soft and hard periodontal tissues accompanied by fixed orthodontic appliances wearing. Finally, the importance of maintenance on patients' health, function, and aesthetics following active therapy was stressed as a priority in the management of both specialties' populations.

Other interesting fields where the interaction between orthodontics and periodontology is very important have not been adequately explored yet. The timing of orthodontic treatment of patient that underwent active periodontal therapy is one area where very little evidence has yet been produced. While most clinicians may agree that orthodontic movement should start after the end of active therapy, there is still no universal protocol that can be applied to patients with periodontally compromised dentition undergoing combined ortho-perio treatment. The influence of the adopted surgical protocol may also have an impact on the timing and regenerative therapies may require longer periods of time compared to traditional periodontal treatments when a translatorily movement direction is required.

Despite the high number of published articles, we realized there is a lack of good evidence about many of the treatments including both orthodontics and periodontal therapy. Well-designed clinical trials evaluating the interaction between these only apparently distant specialties must be encouraged in the dental community. Evaluating patient care from just one specialty eye may limit the possibilities of treatment when compared to a coordinated view of each particular condition. A good perspective can only exist with two points of view.
